# Timing Actions to Avoid Refractoriness: A Simple Solution for Streaming Sensory Signals

**DOI:** 10.1371/journal.pone.0022159

**Published:** 2011-07-15

**Authors:** Javier Nogueira, Ángel Ariel Caputi

**Affiliations:** 1 Departamento de Neurociencias Integrativas y Computacionales, Instituto de Investigaciones Biológicas Clemente Estable, Montevideo, Uruguay; 2 Departamento de Histología y Embriología, Facultad de Medicina, Universidad de la República Oriental del Uruguay, Montevideo, Uruguay; Center for Genomic Regulation, Spain

## Abstract

Segmenting self- from allo-generated signals is crucial for active sensory processing. We report a dynamic filter used by South American pulse electric fish to distinguish active electro-sensory signals carried by their own electric discharges from other concomitant electrical stimuli (i.e. communication signals). The filter has a sensory component, consisting of an onset type central electro-sensory neuron, and a motor component, consisting of a change in the fish's discharge rate when allo-generated electrical events occur in temporal proximity to the fish's own discharge. We investigated the sensory component of the filter by in vitro mimicking synaptic inputs occurring during behavioral responses to allo-generated interfering signals. We found that active control of the discharge enhances self-generated over allo-generated responses by forcing allo-generated signals into a central refractory period. This hypothesis was confirmed by field potential recordings in freely discharging fish. Similar sensory-motor mechanisms may also contribute to signal segmentation in other sensory systems.

## Introduction

Active senses as touch, proprioception, echolocation and electrolocation have sensory receptors tuned to the dynamic range of a self-generated signal carrier. Because such dynamic range may be shared by externally generated events (here referred to as allo-generated), animals have developed different mechanisms to segment self- from allo-generated signals flowing through the same channel.

Some of these mechanisms are based on expectation signals carried by corollary discharges informing the sensory centers about the timing of motor commands and potential consequences of the self-generated actions [1]–[5]. Corollary discharges may be simple facilitatory or inhibitory events occurring at the time of the expected self-generated signals [6]. In other cases, corollary discharges are more complex and plastic, generating mirror images of the precedent sensory flow [3], [7], [8].These images are integrated with the input allowing adaptive filtering of sensory features [7], [8].

Alternatively, descending commands might adapt self-generated actions to the best responsiveness range of a stereotyped sensory filter. Here we show that this principle is applied with maximal economy by pulse Gymnotiforms, a group of weakly electric fish lacking corollary discharges from electromotor commands to early sensory stages [9].

Objects distort the field produced by the electric organ discharge (EOD) and this distorted signal feeds into the electroreceptors on the skin [10], [11]. Electroreceptor afferents project to the electrosensory lateral line lobe where two electrosensory paths (fast and slow) diverge and differ in structure and function [12]–[14].

The slow path arises from electroreceptors sharply tuned to the self-generated EOD. These are innervated by burst–duration-coder primary afferents that projects to the electrosensory lobe [15]. This first sensory relay is a cerebellum like structure in which three somatotopic maps are represented [16]. This complex circuit integrates peripheral electrosensory signals with descending information. It involves many neuronal types organized as intercalated layers of interneurons projecting locally and efferent neurons projecting to superior centers and to the contralateral lobe. It also receives contralateral projection fibers and descending information from electrosensory recurrent loops together with other sensory modalities [14].

The fast path, focus of this study, is characterized by thick primary afferents arising from electroreceptors mainly distributed on the perioral region. These primary afferents discharge a single spike whose latency is inversely proportional to the amplitude of the local stimuli [9], [17]. The first central relay in the fast electrosensory pathway consists of a single type of ‘onset’ spherical neuron that fires a single spike for each electrosensory event [9], [17], [18]. In the somatotopically organized spherical cell layer, local stimulus intensity stimulating individual receptors is encoded as spike firing latency [17]. Thus the electric image is encoded as a somatotopic pattern over the network, in which stimulus intensity is represented as relative spike latency. Spherical neurons project to a mesencephalic nucleus [9], [17], [18] where information carried as spike firing latency appears to be decoded by a coincidence detection circuit, similar to the layer VI of the torus semicircularis of wave type electric fish [19], [20].

In pulse gymnotids, lacking corollary discharge, the presence of regularly occurring allo-generated electric events, as the EODs of nearby conspecifics, may cause ambiguity. Self-generated electric images can be modified by coincidence with external electrical events while intercalated allo-generated images that activate the same electrosensory pathway may be interpreted as self-generated ones. Fish of the genera *Gymnotus* and *Brachyhypopomus* avoid repetitive coincidences of self- and conspecific-EODs by controlling the cycle of the pacemaker driving the electric organ [21]–[24]. Two types of behaviors leading to coincidence avoidance have been observed in these fish: a) in some fish couples both interacting individuals shift the pacemaker rate in opposite direction, similarly to wave type fish [24], b) in other fish couples, pacemakers remain at a similar resting frequency but shift the relative phase between them.

Phase modulations only occur when difference in the mean EOD intervals of both fish is small. The fish discharging at the highest rate tends to accelerate when more than one conspecific EODs precedes the self-generated EOD a few milliseconds [23]–[26]. This behavior is called “jamming avoidance response” of pulse fish after the early description by Westby [23]. Another phase modulation behavior is displayed by the fish discharging at a lower rate. This display was called by Westby “synchronization bouts” [23] and consist of post coincidence accelerations that cause a transient reversion of the rate relationship. Consequently, the instantaneous frequency of both fish oscillates reciprocally around the same value. Interestingly, synchronization bouts in *Brachyhypopomus gauderio* are only present at the mating season [26].

Two interesting problems are raised by phase control displays: a) how fish detect imminent or actual EOD coincidence and b) what are the sensory consequences of these responses beyond coincidence avoidance.

The first problem is still a matter of debate and out of the scope of this article. Adaptive responses at primary afferents [27]–[28] and at the electrosensory lobe [29] have been postulated. This article focuses on the sensory consequences of coincidence avoidance behaviors at the fast electrosensory pathway. In this path the intercalated self- and allo- generated spikes fired by an onset neuron convey the information on self-generated images originated from passive objects and communication signals originated by conspecifics. Thus avoiding coincidences does not rule out interference because the two images (self- and allo-generated) are simultaneously encoded in the same physical channel.

Having in mind some known features of the sensory [9], [18] and motor [21]–[26] branches of the active electric sense we conducted experiments to evaluate the consequence of interference signals in the reliability of self-generated signals. We aimed at: a) characterizing the phase relationship between self- and allo-generated events during behavioral displays; b) characterizing the excitability of the fast electrosensory onset cell type at the electrosensory lobe; and c) showing how these two features may be combined to sort out the signals from different origins.

## Results

### Phase relationship between self- and allo-generated events during behavioral displays

**Figure 1:**
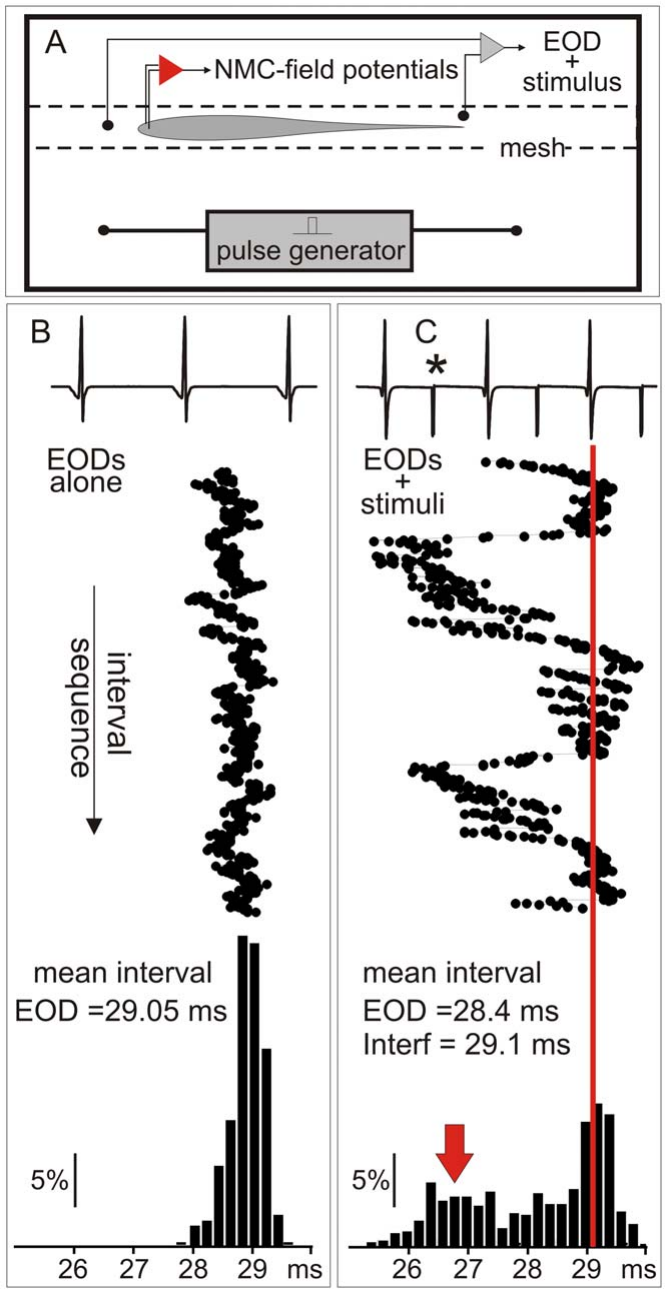
The time series of EOD in resting and under interference in *G. omarorum*. **A**) The experimental setup for behavioral experiments consisted of a small tank where the fish was restrained by a net. Interference stimuli were square pulses delivered to the water at a constant rate. The head to tail EOD and the interference artifact were recorded. In some experiments brain field potential at the magnocellularis mesencephalic nucleus were recorded (represented by the amplifier in red). Inter-EOD intervals sequences are compared in B (resting condition) and C (under interference). B) Top: raw data obtained from the head to tail EOD recordings at rest. Middle: Raster plot of the inter EOD intervals. Bottom: Histogram of the first-order inter-EOD-intervals. C) Top: raw data obtained from the head to tail recordings showing the EOD and the interference artifact (asterisk). Middle: Raster plot showing transient shortenings of the inter EOD intervals under interference condition. Bottom: Histogram of the first-order inter-EOD-intervals showing two modes, one corresponding to longer intervals and the other (red arrow) corresponds to the largest accelerations.

**Figure 2:**
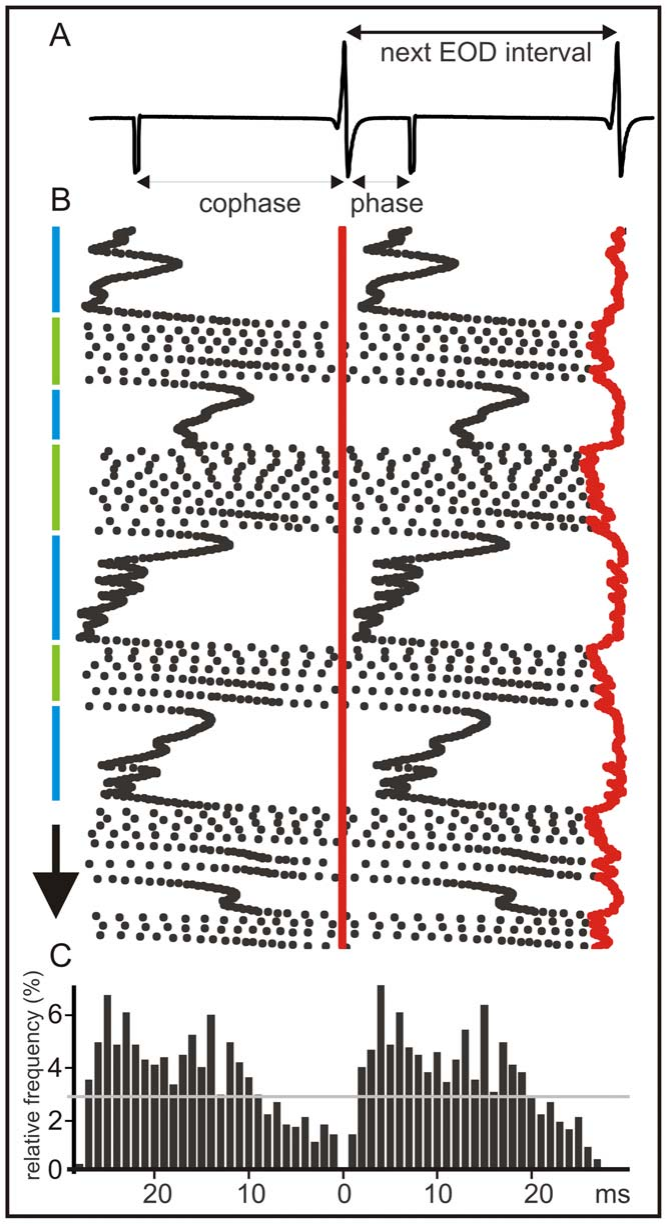
Coincidence avoidance behavior of *G. omarorum*. The example shows a typical display elicited by artificial electric pulses (1 ms duration) delivered at a constant frequency at about the mean EOD rate. The raster plot is referred to the EOD timing (the vertical series of red dots). A) Raw data showing the EOD and the interfering stimuli artifacts. The following terms are defined: a) inter EOD interval (time between successive EODs), b) interference stimulus interval (time between successive stimuli) which is the sum of c) phase (time between EOD and next interference stimulus) and d) co-phase (time between interference stimulus and next EOD). B) Raster plots showing the time course of the inter-EOD interval (red dots) and the time course of the phase (black dots to the right of the EOD) and the co-phase (black dots to the left of the EOD). The arrow indicates the course of the experiment (total duration 30 s). Series of transient acceleration responses were triggered by interfering stimuli preceding the pacemaker discharge by less than 5 ms [21]–[27]. This behavioral display occurred when pacemaker rate is slightly faster than the interference (jamming avoidance, green segments on the side). During these periods the phases of the interfering stimuli progressively increase up to about 20 ms, showing a slight trend to be phase locked at about this latency. Beyond this point the phase increases at a faster rate due to the acceleration of the pacemaker. Pacemaker accelerations were also triggered by coincidence between EODs and allo-generated events when the EOD rate is slower than the rate of interfering pulses (synchronization bout [24]–[27], blue segments). C) Peri-EOD histogram showing that the probability interfering pulses phases (black bars to the right of the reference point) and co-phases (black bars to the left of the reference point). Note that the probability is smaller than average at a late phases (dashed line), but larger than average at earlier phases.

Phase modulation displays are characterized by sudden increases in the pacemaker rate. We reproduced these behavioral displays by delivering a train of artificial stimuli (interfering stimuli) at a constant frequency, similar to the fish's own discharge rate (Fig. 1 and 2). The raster plots of Figure 1 compare the inter EOD interval sequence in the presence and the absence of interference. Under interference, transient accelerations of the EOD rate occur. They are represented as a second peak of the first-order inter-EOD histogram (Fig. 1C bottom, red arrow).

Figure 2 illustrate a typical behavioral experiment. For almost all EOD intervals there is an interfering intercalated stimulus (Fig. 2A). A combined raster plot show how fish alternate jamming avoidance responses (blue segments, Fig. 2 B) and synchronization bouts (green segments, Fig. 2 B). The raster plot is referred to the EOD timing (the vertical series of red dots). The next EOD is represented by the red series of dots on the right side. The interval between the reference EOD and the following (phase) and previous (cophase) interference (black dots) varies due to the change in the EOD instantaneous intervals.

Fish displays provoke an uneven phase distribution of allo-generated events around the EOD as it is shown by the peri-event histograms (Fig. 2C). This is different to what would be expected for two independent clocks. As a consequence of fish displays the number of allo-generated events at short phases after the EOD is increased at the expense of the number of allo-generated events at longer phases. A second less marked peak appears at about 15–20 ms after the self-generated EOD. While the first peak is due to the synchronization bouts, the second is associated to jamming avoidance responses. Both displays cause a reduction of the probability of interfering stimuli occurrence before the next self-generated EOD (Fig. 2B).

Considering that in all our experiments the intervals between allo-generated stimuli were constant, peri-stimulus intervals indicate that the probability of interference at different phases along the inter-EOD interval is highly dependent (and to some extent controlled) by fish's behavior.

### The dynamic filter hypothesis

The fine characteristics of the electric image decoder at the magnocellularis nucleus are still unknown. However, this does not preclude asking whether the onset cells respond differently to each of the intercalated patterns of stimulation. Thus, we analyzed the responsiveness of either individual cells or the whole sensory path to each one of the series (EOD and interference timed) of stimuli.

Taking into account the changes in EOD sequence occurring in the presence of interference (Fig. 1), the peri-EOD probability pattern of interference caused by these behavioral displays (Fig. 2) and the long refractory period of spherical cells [18], we inferred that the pacemaker responses to sensory interference cause significant differences between the responses of spherical neurons to EOD and to interfering stimuli. We tested the hypothesis that the interplay of the central regulation of the pacemaker and the intrinsic properties of spherical neurons acts as a dynamic filter that gates the self-generated signals to the detriment of interfering events. We provide two types of evidence: i) *In vitro* studies showed firstly that a low threshold K^+^ current dominates spherical neuron excitability determining a long lasting refractory period. Secondly, the analysis of data obtained by intercalating EOD- and interference-timed stimuli as observed in a behavioral experiment showed that spherical cell's responsiveness was greater when stimuli were timed to mimic the EOD than when stimuli were timed with the interference, ii) *In vivo* studies showed that the reliability of the signals carried by the fast electrosensory pathway in response to self-generated EODs is larger than the extracted from interfering artificial pulses. Next these evidences will be described more thoroughly.

### *i1) In vitro* studies, spherical cell responsiveness

**Figure 3:**
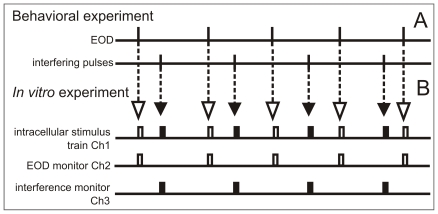
Construction of the intracellular stimulus train. A) Relative timing of EODs and interfering events were selected from behavioral experiments files using a time resolution of 20 microseconds. From these data we constructed a file having three channels (in house software): Channel 1) the intracellular stimulus train, in which EOD-timed stimuli consisted of a rectangular pulse of 1.5 ms duration starting at the timing of the EOD (open rectangles) and the interference-timed stimuli consisted of a rectangular pulse of 1.5 ms duration starting at the timing of the interfering event (filled rectangles); Channel 2) the EOD timing monitor in which only EOD-timed stimuli were represented; and Channel 3) the interference timing monitor in which only interference-timed stimuli were represented. Channel 1 was used to drive the step activation port of the Axoclamp 2B. Intensity was controlled using Axoclamp 2B step activation controls. Channels 2 and 3 were recorded simultaneously with the voltage and current outputs of the Axoclamp 2B.

**Figure 4:**
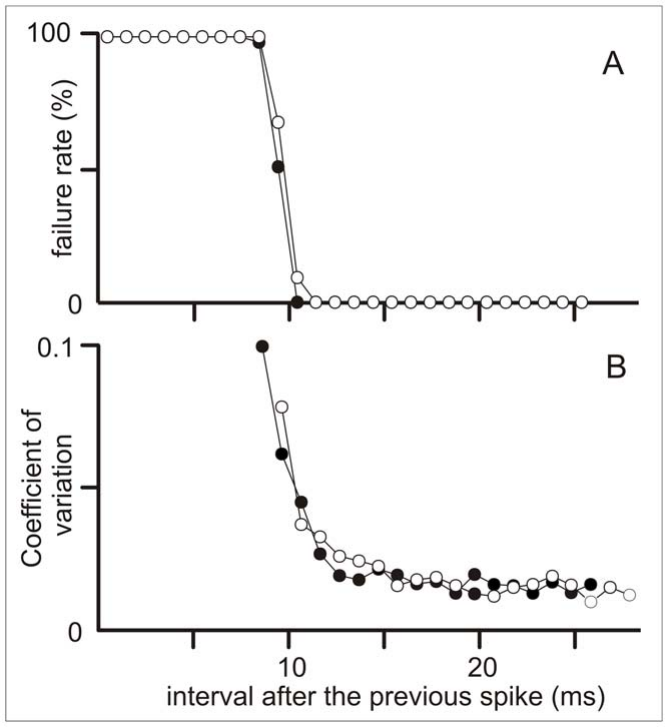
Spherical cell refractoriness. When the cell received stimulus patterns containing the series of intervals obtained during behavioral experiments the refractory period following any spike (either elicited by the EOD timed stimuli, open symbols, or interference timed stimuli, filled symbols) was identical: A) absolute refractory period (stimulus failure rate vs. previous inter-stimulus interval); B) relative refractory period (coefficient of variation of spike latency vs. previous inter-stimulus interval).

Fifteen spherical neurons were visually identified in brain slices and recorded in whole-cell, current-clamp mode. We first explored the excitability of 9 cells under natural patterns of stimulation. To mimic the synaptic afferent input that the neuron would have received during a coincidence avoidance behavioral display, we used a stimulus regime reconstructed from previous behavioral experiments (Fig. 3). We mimicked the effects of the EODs and the allo-generated stimuli using identical depolarizing pulses intercalated according to pre-recorded behavioral patterns (1.5 ms, 5–50% above threshold, Fig. 3). Under this natural stimulus sequence we confirmed our previous report of long lasting refractoriness [18]. Refractoriness was characterized by plotting failure rate (Fig. 4A) and coefficient of variation of the spike latencies (CV, Fig. 4B) as a function of inter-stimulus interval. When the stimulus train was only the train of stimulus timed with the EOD, the intervals were always longer than 25 ms and consequently failure rate was null. When EOD- and interference-timed stimuli were intercalated, any stimulus preceded by a spike for less than 12–14 ms, either did not evoked a spike (Fig. 4A) or increased the spike latency variability (Fig. 4B). Based on the reduction of cell responsiveness to the same identical stimulus in the presence and absence of interference, we conclude that the upper limit of overall mutual information between input and output of individual cells should be reduced by the presence of interference [30].

**Figure 5:**
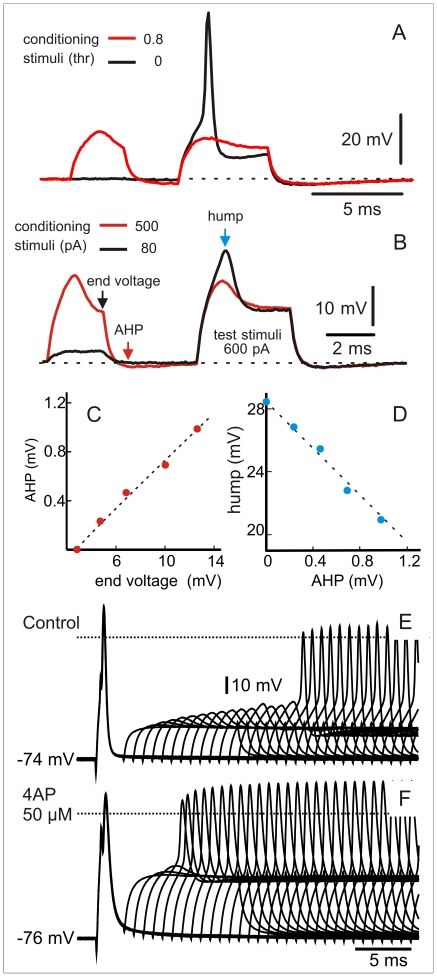
A low threshold K^+^ current determines the long refractory period. A) Sub-threshold conditioning stimulus (red trace, 0.8 thresholds) prevent neuron spiking in response to test stimuli. Spike is elicited by a control identical test stimulus in the absence of a conditioning one (black trace). B) Paired pulse stimulation with sub-threshold stimuli show that depolarization leads to an hyperpolarization after the end of the pulse (red arrow)and a decrease of excitability of the neuron evidenced by the reduction depolarization peak in a constant amplitude test pulse (blue arrow). Linear relationships between end conditioning depolarization (end voltage-black arrow), after-hyperpolarization (red arrow) and the amplitude of the hump evoked by the test pulse (blue arrow) are shown in C and D). E) Pair pulses applied at different delays shows a long lasting refractory period. F) As expected for a low threshold K^+^ conductance, refractoriness was shortened after the application of 50 microMolar 4-aminopyridine to the bath.

In other six cells we explored the underlying mechanism of long lasting refractoriness. In all these cells, spike firing was followed by the activation of a low threshold, non-deactivating, conductance that prevents repetitive firing. This conductance was activated at sub threshold intensities (Fig. 5A to D) and endowed the spherical cell with one of the most elemental and powerful neural filters: a post spike reduction of excitability lasting between 10 and 15 ms (Fig. 5D and E). The application of 50 micromolar 4-aminopyridine (4-AP) to the bath always shortened, between one half and two thirds, the duration of the refractory period (Wilcoxon sign-rank test, N = 6, p = 0.031, Fig. 5F). The appearance of the described long refractory period after sub-threshold conditioning stimulus and the reduction of its duration by small doses of 4-AP, indicates that it is mainly due to a low threshold K^+^ conductance.

### *i2) In vitro* studies, onset type neurons responsiveness depends on stimulus origin

Data from the 9 experiments performed intercalating EOD- and interference-timed stimuli were re-analyzed by selecting peri-stimulus epochs (from 1 ms before to 5 ms after each stimulus) from the continuous membrane potential trace. These epochs were classified in two series labeled as responses to EOD-timed or to interference-timed stimuli and responsiveness to both types of stimuli were compared.

**Figure 6:**
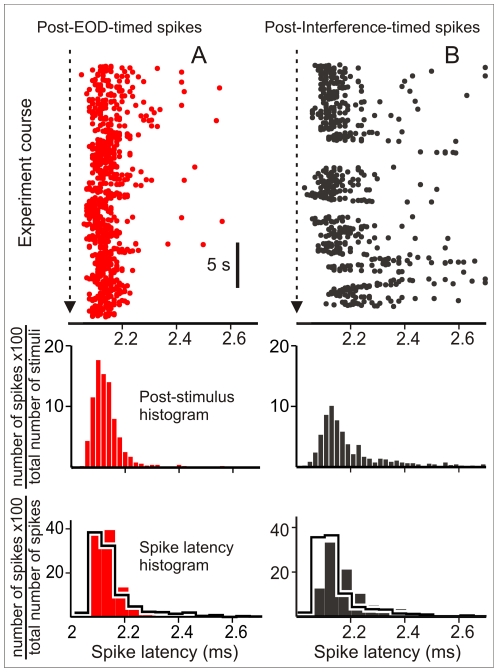
Interference and EOD timed stimuli are not equally effective. A) Top: Post stimulus raster plot of the spiking responses to EOD-timed pulses applied as in the natural sequence (red dots). Middle: corresponding post stimulus histogram (ordinate: 100× EOD evoked spikes per bin divided total number of EOD-timed stimuli, red bars). Bottom: The responsiveness to the natural patterns was compared with the responsiveness to the same set of intervals applied in a shuffled sequence in a pair wise experiment (histograms in heavy black lines). For the sake of clarity spike latency histograms showing the relative frequency of the latency after the cell spiked (ordinate: 100× EOD evoked spikes per bin divided total number of EOD evoked spikes) were constructed with a different bin size. B) Top: Post stimulus raster plot of the spiking responses to interfering pulses timed as in the natural sequence (black dots). Middle: corresponding post stimulus histogram (ordinate: 100× interference evoked spikes per bin divided total number of interference-timed stimuli, black bars) Bottom: The responsiveness to the natural patterns was compared with the responsiveness to the same set of intervals applied in a shuffled sequence in a pairwise experiment (histograms in heavy black lines). For the sake of clarity spike latency histograms showing the relative frequency of the latency after the cell spiked (ordinate: 100× interference evoked spikes per bin divided total number of interference evoked spikes) were constructed with a different bin size. In the natural sequence experiments the area (stimulus effectiveness) and the sharpness of the spike latency histograms (latency precision) in response to EOD-timed-stimuli (A, red post-stimulus histograms) were larger than those corresponding to interference (B, blue post-stimulus histograms). In the pair wise shuffled-EOD experiment the histograms are similar to each other (superimposed histograms in heavy black lines in the bottom panels of A and B).

Failure rate was smaller for EOD-timed stimuli than for interference-timed stimuli in all runs (14±3.7% and 36.7±4.1% for EOD-timed and interference-timed stimuli respectively, Wilcoxon signed-rank test p<0.01, N = 11 epochs in 9 cells stimulated at 1.1±0.02 times thresholds). In addition, the latencies of spikes evoked by EOD-timed stimuli were less variable than the latencies of the spikes evoked by interference-timed stimuli (CV 0.043+0.01 vs. 0.069±0.015 for EOD-timed and interference-timed stimuli respectively, Wilcoxon signed-rank test p<0.01, N = 11 epochs). Post stimulus raster plots and their corresponding post stimulus histograms illustrate these differences in cell response (Fig. 6A to D). In addition normalized spike latency histograms (Fig. 6 E and F) show a different distribution of spike latencies evoked by both trains.

Taking into account that all stimuli were identical, the differential effect on the spike failure rate and latency variability evoked by the EOD and the interfering stimuli can only be attributed to the pacemaker phase modulation. Responsiveness dependence of the sequence of the events was confirmed by control experiments (Fig. 6E and F superimposed histograms in heavy black lines). In 6 neurons, responses to the natural stimulus series were compared with responses to a control series composite of the same subset of EOD-timed stimulus intervals but applied in a shuffled order. Differences in the post stimulus histograms obtained for spikes evoked by EOD-timed and interference timed stimuli disappear in EOD shuffled stimulus runs. Statistical comparisons showed: a) significantly smaller failure rates and spike variability for EOD-timed stimuli in the natural than in the shuffled interval series (ranked-sum test, p = 0.0022, 8 epochs, 6 cells); b) significantly higher failure rates for interference-timed stimuli in natural patterns than in shuffled patterns (ranked-sum test, p = 0.0022, 8 epochs, 6 cells); c) no significant differences between shuffled control patterns (failure rates = 28.9±7.5% vs. 28.8±4.8%; and CV = 0.062±0.014 vs. 0.059±0.009 respectively; Wilcoxon signed-rank test, p>0.1, 8 epochs, 6 cells).

**Figure 7:**
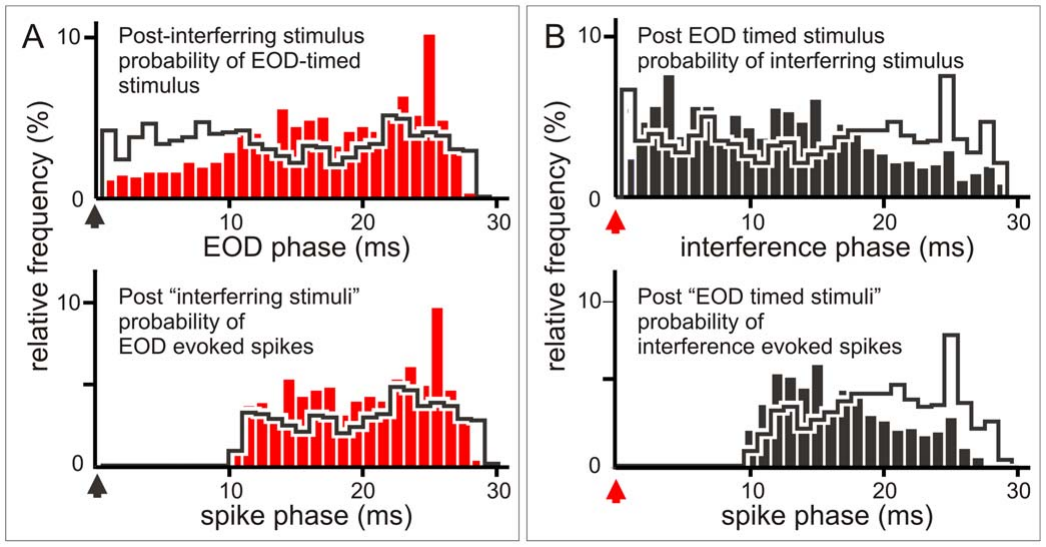
Refractoriness and post-stimulus probability explain the pattern dependent differences in responsiveness. A) Top row: Post-stimulus histograms showing the peri-interference (black arrowhead at 0) histogram of the EOD (red bars) and the shuffled EOD timed stimuli (histograms in heavy black lines). Ordinate: 100× number of EOD per bin divided by the total number of interference timed stimuli. Note the smaller EOD timed stimulus probability at the earlier phases and the slightly larger at longer phases. Bottom: Post-interference histogram of EOD evoked spikes. Ordinate 100× number of EOD evoked spikes per bin divided by the total number of interference-timed stimuli. Because of the absolute refractory period stimuli at earlier phases are similarly blocked in experiments made with natural and shuffled time series. However, since the probability of stimuli at earlier phases is the lowest for EOD timed stimuli, this sequence is the less affected by refractoriness. B) Top row: Post-stimulus histograms showing the post-EOD (red arrowhead at 0) histogram of the interference (black bars) and the shuffled interference timed stimuli (histograms in heavy black lines). Ordinate: 100× number of interference-timed stimuli per bin divided by the total number of EOD-timed stimuli. Note the slightly larger interference timed stimulus probability at the earlier phases and the clearly smaller at longer phases. Bottom: Post-EOD histogram of interference evoked spikes. Ordinate: 100× number of interference evoked spikes per bin divided by the total number of EOD-timed stimuli. Since the probability of stimuli at earlier phases is the highest for interference timed stimuli, this sequence is the most affected by refractoriness. Thus, EOD-timed stimuli were more efficient because natural pacemaker modulations kept them out of the window of refractoriness following responses to interference. In addition, pacemaker modulation kept most of the interference stimuli in the window of refractoriness following the EOD. Therefore, a larger proportion of interference timed stimuli either was blocked or evoked spikes with more variable timing.

Differences in failure rates and latency variability can be easily explained by weighting the refractoriness effect with stimulus probability. Pacemaker modulation determines that the probability of the interference-timed stimulus falling within a window of 12 ms after the EOD-timed stimuli was always much larger than that of the EOD-timed stimulus falling within the same window after the interference timed stimuli (Fig. 7A and B). Due to the differences in phase probability, the absolute refractory period (phase below 10 ms) causes a larger amount of failures to interference-timed stimuli (Fig. 7C and D). Note that the probability of completely blocked stimuli at phases below 10 ms is much smaller for EOD-timed than for interference- timed stimuli (Fig. 7). In addition, due to the spike latency variability caused by the relative refractoriness and the larger amount of interference evoked stimuli at phases between 10 and 15 ms, the reliability of latency for faithfully representing the stimulus is decreased. Therefore, as a consequence of refractoriness and phase distribution, latency reliability for a given applied stimulus intensity is larger for the EOD-timed train than for the interfering stimuli. Moreover, peri-event histograms also show that the probability of interference-evoked spikes occurring at long delays after an EOD (i.e. risking interference with the up-coming EOD) is lower than the probability observed for the same delays in the control experiments (compare black bars with histograms in heavy black lines, Fig. 7 D) because the fish accelerates its own discharge rate in the presence of a stimulus. This acceleration determines a faster phase advance and, subsequently, a drastic phase reduction when the inter EOD interval falls entirely within the allo-generated interval. This reduces the number of EODs jammed by the refractory period of spikes evoked by a preceding allo-generated stimulus. This effect is not observed for interference-timed stimuli (compare red bars with histograms in heavy black lines, Fig. 7 C).

**Figure 8:**
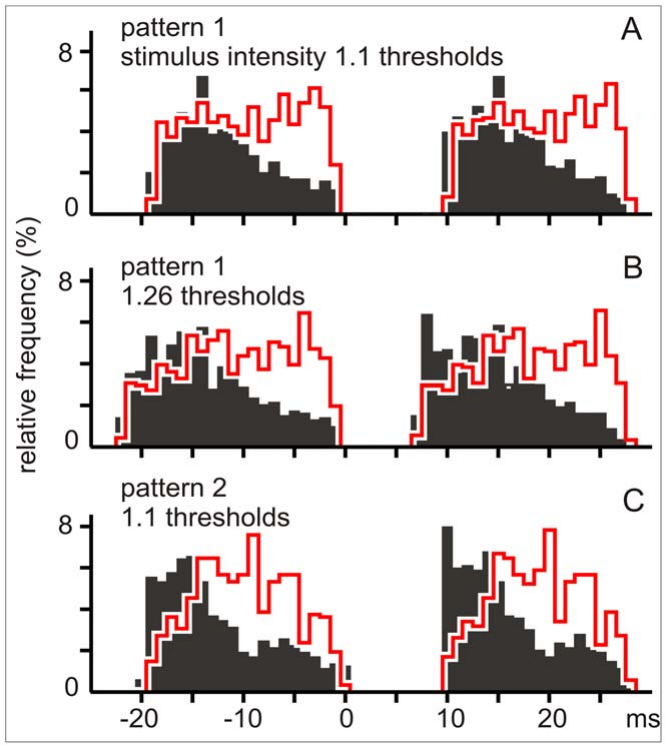
Robustness. The post-stimulus histograms of evoked spikes illustrate one of the experiments in which the same neuron was stimulated with different intensities (1.1 and 1.26× threshold in A and B respectively) or by two sequences obtained from different behavioral experiments (compare peri-event histograms A and C obtained with 1.1× threshold stimuli). Black bars correspond to the probability of interference evoked spikes after the EOD timed stimuli. Histograms in heavy red lines correspond to the probability of spikes evoked by EOD timed stimuli after the interference timed stimuli. For the black bars ordinate represents the number of interference evoked spikes per bin divided by the total number of EOD-timed stimuli. For the heavy red line histograms ordinate represents the number of EOD evoked spikes per bin divided by the total number of interference-timed stimuli.

The robustness of the phenomenon was tested by applying to the same neuron a) different stimulus trains reconstructed from two different behavioral experiments (2 cells) and b) the same train at different intensities (4 cells). Neither the intensity nor the particular behavioral display affected the differential effects of interval sequence of the pulses mimicking self-generated signals (Fig. 8).

### *ii) In vivo* experiments, path responsiveness in the freely discharging fish facilitates self-generated signals

Field potential responses of the fast electrosensory path are composite spikes, whose amplitude and sharpness result from the degree of coincidence of all spikes fired by the population of spherical neurons. Consequently, since the spike latency is the encoding variable, the composite spike stability for a constant input is a good index of signal reliability throughout this path.

**Figure 9:**
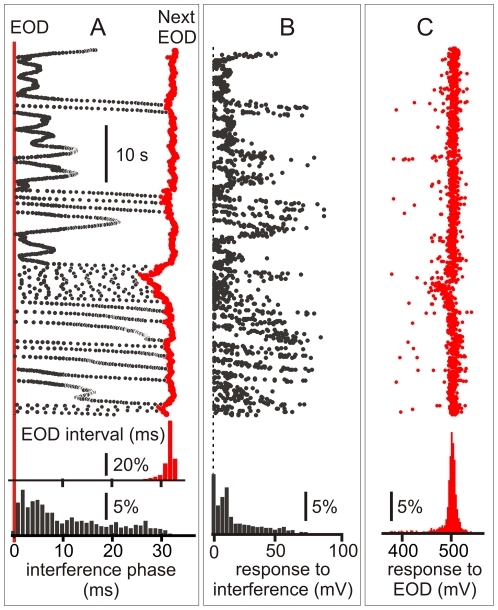
Field potential responses to self- and allo-generated signals in freely discharging fish. A) The raster plots shows the time course of phase of interfering stimulus (black) and the next EOD interval (red) in a freely discharging fish chronically implanted at the magnocellularis nucleus. Histograms at the bottom in show the relative frequency of interference after the EOD (black bars, ordinate: 100× number of allo-generated stimuli per bin divided the total number of EODs) and the first order interval histogram (red). B) Raster plot representing the amplitude (abscissa) of the field potentials evoked by the EOD at the magnocellularis nucleus (the time course of the experiment in register with A) and the amplitude histogram. C) Raster plot representing the amplitude (abscissa) of the field potentials evoked by the EOD at the magnocellularis nucleus (the time course of the experiment in register with A and B) and the amplitude histogram. Note that amplitude histogram in B shows a relatively larger dispersion than in C.

In vitro results suggested that field potentials responses to the EOD would be less variable (hence a better representation of the input) than responses to interfering stimuli. This prediction was confirmed in two fish chronically implanted with recording electrodes at the projection nucleus of the spherical neurons (see Methods). Implanted fish displayed coincidence avoidance patterns when a train of electric stimuli of constant frequency was applied to the water at about the rate of the EOD. In epochs where the fish had a constant spatial relationship to the stimulation electrodes and the tank, the composite spike evoked by the EOD was relatively stable in shape and amplitude but the composite spike evoked by the interference was several times more variable in both animals (Fig. 9). These data and previous arguments lead us to conclude that this mechanism serves to enhance self-generated signals to the detriment of interference.

## Discussion

### Active sensory gating of self-generated signals

In many sensory systems segmentation of self-generated signals are based on expectation mechanisms implemented by internal commands either facilitating desired signals or inhibiting undesired signals. These mechanisms are implemented by synaptic control of sensory cells response. Here we report that sensory gating can also be implemented by the intrinsic neuronal properties if motor actions are timed in a proper way. Our findings stress the crucial role of the intrinsic properties of onset neurons in sensory processing providing an additional example of the general rule that “auto rhythmic electrical properties of central neurons and their connectivity form the basis for an intrinsic functional coordinate system that provides internal context to sensory input” [31].

We report a dynamic filter having a sensory component, based in the intrinsic properties of an onset type central electro-sensory neuron, and a motor component, based on reflex accelerations of a command nucleus consisting on tightly coupled pacemaker cells. Pacemaker accelerations are triggered by allo-generated electrical events occurring in temporal proximity to the fish's own discharge. As a result of accelerations self-generated signals are facilitated to the detriment of allo-generated ones.

This is an alternative mechanism to the tonic shift in EOD frequency observed in wave fish (jamming avoidance response) [32], [33]. Wave fish strategy reduces the amplitude and phase modulation of the self-generated signal. The transient pacemaker accelerations exhibited by pulse gymnotiforms force allo-generated signals into the onset neurons refractory period after their self-activation. Although the decoder properties of the next relay are still not well known, our findings suggest that the accuracy of the spike timing for faithfully representing the stimulus intensity is less for allo than for self-generated signals. This suggests a differential effect of fish behavior on information transmission for self- and allo-generated signals.

The observed differences in spike failure rate and latency variability appear to be small at the single cell level (22% in failure rate and 2.5% in variability). However, it should be noted that the image of a conspecific is composed by local stimuli of smaller amplitude and distributed over a smaller area of skin than the basal self-stimulation. As a consequence only a fraction of the onset cell population receives the input of the allo-generated stimuli. In addition, it is generally thought that signals are decoded at the magnocellularis nucleus by coincidence detection [19], [20], [34]. This mechanism is impaired either by the lack of arrival or by the arrival out of time of an afferent impulse. The enhancement of the differences in responsiveness by a population effect may strength the power of the sensory filtering. This is compatible with the results obtained in chronically implanted animals.

One interpretation of the described mechanism is that the fish changes its frequency to keep the allo-generated signals in the “refractory shadow” of its own responses. This behavioral display called “echo response” actually occurs in Mormyrid fish, whose EOD rate is slower and use corollary discharge for segmenting self-generated signals [35]. This behavior requires matching the external EOD frequency as the best strategy. Instead, in *Gymnotus omarorum*, the reduction of the jamming effect is a consequence of pacemaker responses to sensory interference. In fact, theoretical and experimental studies [24], [26], [35] suggest that it is not necessary for the fish to identify a real “self-generated” signal to generate the avoidance behavior. These studies [24], [26], [36] indicate that detection of a few novel stimuli is enough to trigger the jamming avoidance behavior. Accelerations are also triggered by stimuli either shortly preceding or coinciding with a substitute EOD in a curarized fish [26]. It can be conceived that the same sensory mechanisms used for novelty detection may also serve for triggering the accelerations necessary for a mechanism of sensory filtering by motor timing. Although less efficient than a precise prediction of the timing of the interference, reacting to novelty appears to render efficacy and safety. Transient accelerations are a common response of electric fish to changes in self-generated signals (so called novelty responses) [10], [37]. These responses correspond to the detection of differences between the present electric image and the moving average of between 100 and 1000 previous ones [37]. It is likely that the interference either by coincidence or by affecting the responsiveness of the slow sensory path may be involved in triggering pacemaker accelerations.

### Comparative considerations

Onset cells are characteristic of the fast auditory paths of higher vertebrates [38,39,40] and the electrosensory path of gymnotiform fish [18]. The presence of a low threshold slow inactivating K^+^ channel endows onset neurons with its single spiking characteristics. Comparative data suggest that onset cells are a convergent solution in the evolution of time coding sensory systems [41]. However, species variations may adapt cell structure and specific functions. The membranes of some second order neurons of auditory [42], [43] systems and electrosensory [18] systems are relative clamped at rest by the presence of the low threshold K^+^ conductance (reducing depolarizing responses) and by a mixed cation conductance (Ih, reducing long lasting hyperpolarization and facilitating rebound responses [39], [43], [44], [45]). By making the membrane most sensitive to fast large transients, these channels support signal encoding by the precise timing of the spikes. The intrinsic properties of onset cells in upstream centers (nucleus laminaris of birds [40], [42], medial superior olive of mammals [43], [44] and possibly in the magnocellularis nucleus of *Gymnotus* [36] help them to work as precise coincidence detectors. In fact it has been reported that the partial blockade of low-threshold potassium channels reduced coincidence detection by slowing the rise of current needed to evoke a spike [43]. This may happen in spherical cells of the lateral map receiving multiple club endings [9], [12]. It is clear from Fig. 3 that, even after a sub-threshold depolarization, the activation of a low threshold K^+^ channel closes the detection window briefly after the beginning of the depolarization, thus facilitating coincidence detection.

Some onset auditory cells exhibit another characteristic here shown as exploited by the nervous system for signal integration: a long refractory period. The low-threshold K^+^ channel observed in auditory cells of guinea pig [39] deactivates slowly, as does the K^+^ low-threshold conductance described for pulse fish spherical cells [18]. To our knowledge the computational role of refractory periods in the onset cells of the auditory system is not well characterized yet. In other auditory cells, low-threshold K^+^ conductance is supplemented by the presence of a high-threshold K^+^ conductance that facilitates spike repolarization and accelerates deactivation of the low threshold current, allowing the auditory onset cells to follow high frequency stimuli [38], [39], [41].

In spinal circuits of the somatosensory system the presence of long refractory periods that control the relative timing of self- and allo-generated stimuli may be a simple and efficient mechanism to facilitate (or to block) either self- or allo-generated signals. In fact, all the necessary elements appear to be present at least in the somatosensory system. It has been proposed that motor timing of self-generated actions may be centrally regulated by discrete descending commands [46]. It has also been shown that this self-stimulated rhythm may be transmitted to sensory afferents [47], [48] and that some neurons of the dorsal horn express a low threshold K^+^ conductance [49]. Thus, a mechanism to avoid refractoriness could be present.

### Conclusions

We are showing that a low threshold K^+^ current of a second order neuron and the reflex control of a rhythmic pacemaker nucleus are necessary conditions that, if combined, gates self-generated signals present in a mixed train of self- and allo-generated sensory events. Thus in the presence of interference, this mechanism facilitates self-generated signals in such a way that superior centers receive a steadier stream. Comparative analysis indicates that the widespread type of vertebrate neurons referred to as a single spiking onset phenotype is based on a similar repertoire of ionic conductances. In some systems the role of onset neurons intrinsic properties on timing precision and coincidence detection has been emphasized. Here we introduce a new computational role based on the long duration of their refractory period.

## Materials and Methods

All experiments were performed in fish of the species *Gymnotus omarorum* [50] ranging between 12 and 18·cm in length. This species is easily gathered in lakes and creeks close to Montevideo (latitude 35.5°, longitude 55°).

### Ethics statement

Experiments were approved by the Animal Ethics Committee of the Instituto de Investigaciones Biológicas Clemente Estable (protocol number 001/03/2011). Fish care and experiments were performed under the regulations of the Comisión Honoraria de Experimentación Animal of the Universidad de la República (ordinance 4332-99) and the International Guiding Principles for Biomedical Research Involving Animals. All surgical procedures were performed under deep anesthesia induced by pentobarbital (0.5 mg i.p.).

### Behavioral experiments

We elicited a coincidence avoidance behavior similar to that described by Westby [19]–[21] in three intact fish. Interfering stimuli (squared pulses of 1·ms duration) were generated by an electrical stimulator, optically coupled through a battery-operated stimulus isolation unit that fed two electrodes placed 10·cm apart on a line para-sagittal to the fish. For each trial, stimulus frequency was set at the mean EOD rate and maintained constant throughout the recording epoch. Stimulus amplitude was manually adjusted until the fish displayed the required behavior. The EODs and the artificial stimuli were electronically separated using a Schmitt trigger and their timings were independently recorded in separated channels. We visualized the data using raster plots referred to the EOD and the interfering pulses and estimated the peri-EOD and peri-interfering pulses probability using relative frequency histograms (Fig. 1).

### Intracellular recordings of spherical cells

Brain slices transverse to the main axis of the brain stem were obtained using a vibro-slicer. Slices were incubated in a low sodium solution containing (mMol·l^−1^): KCl (2), CaCl2 (2.6), KHPO4 (1.25), NaHCO3 (24), MgSO4 (1.6), glucose (20), NaCl (0) and sucrose (201), pH·7.4. After a period of 30–60·min, slices were transferred to the standard recording solution of the same composition but lacking sucrose and having a physiological concentration of NaCl (120·mMol·l^−1^). Spherical cells are located in a monolayer at the border between the deep neuropil and granule cell layers of ELL and could be identified using Nomarski optics under infrared illumination. Whole cell patch recordings were obtained using 6–12·MOhms tip-polished microelectrodes filled with a solution containing the following (mMol·l^−1^): potassium gluconate 122, MgCl2 2.5, magnesium gluconate 5.6, CaCl2 0.3, Na2ATP 5, K-Hepes 5, H-Hepes 5, EGTA 1, pH·7.4.

Spherical cells were intracellularly stimulated with trains of identical rectangular pulses (Fig. 3). To construct these trains we used 3 epochs from a bank of behavioral experiments (30 s duration, Fig. 1). The stimulus file consisted of a series of identical pulses (1.5 ms in duration) applied at the time of the EOD (open rectangles) or at the time of the interfering pulse (filled rectangles) obtained from behavioral experiment files. We recorded four simultaneous channels : a) the current and b) voltage outputs of the Axoclamp 2B used for intracellular stimulus and membrane potential recordings and two separated images of each of the subset of pulses (EOD- and interference- timed, c and d respectively). These signals were digitized with a Digidata 1322A and post processed using MATLAB. After the experiment, peri-stimulus epochs (from 1 ms before to 5 ms after each stimulus) were selected from the continuous membrane potential trace using an in-house software. These epochs were classified in two series labeled as responses to EOD-timed or to interference-timed stimuli. In the very few cases of coincidence we assigned the stimulus pulse to the earliest event. For each stimulus, we detected a spike when a fast transient in the membrane potential caused by the stimuli crossed a depolarization level of −20 mV. Spike latency was defined as the interval between the current step onset and the level crossing time of the spike. For each 6 ms frame of each series we checked that the selected detection level was suited to select all visually discriminated spikes. Because these cells fire a single spike and relative latencies are compared upstream in the path by a coincidence detection circuit with a resolution of less than 1 µs it is generally assumed that electric images are encoded as a somatotopic firing pattern over the network, in which carrier modulation over the skin is represented as relative spike latency at the terminals of the axon of spherical cells neurons. The probability of response and the spike latency distribution were calculated for each series. We measured and compared message uncertainty using two indexes: the failure rate and the coefficient of variation of the spike latencies (CV) when the cell was facing identical stimuli. In both cases smaller indexes indicate a larger reliability of the neural representation of the electric images. To further pinpoint the role of the sequence of intervals between stimulus belonging to one and other series, we performed 8 pair wise control experiments in 6 cells (2 patterns in 2 of the cells plus 1 pattern in the other 4 cells, stimulus intensity 1.1 threshold in all 8 comparisons) where stimulus series obtained from the natural behavior were followed by a control series in which inter-EOD intervals were shuffled. These 8 cells were stimulated with two stimulus trains based on behavioral data: a) a train of identical pulses timed as the recorded EOD and the intercalated, constant frequency interfering signal; and b) a train of pulses containing the same inter-EOD intervals randomly shuffled, together with the intercalated constant frequency interfering signal. After the experiment, peri-stimulus epochs (from 1 ms before to 5 ms after each stimulus) were selected from the continuous membrane potential trace. To test whether the results were affected by the specific behavioral pattern we used three different patterns obtained from different behavioral experiments (pattern 1 = 4 cells; pattern 2 = 4 cells, and pattern 3 = 3 cells; pattern 1 and 2 were played on the same cell in two cases). To test whether the differential effect of the interval sequence was valid for a range of stimulation intensities we played the same natural patterns at different intensities (ranging from 1.05 to 1.5 thresholds) in 4 cells. In two other cells we compared the effects of 2 different intensities (1.1 and 1.26 thresholds) for each of the two different natural patterns played into the cell. In 6 other spherical cells paired pulse stimulation using different delays was used to evaluate the duration of the refractory period before and after the application of 4-aminopyridine (50 microMolar) to the bath.

### Field potential recordings in vivo

The activity evoked at the magnocellularis mesencephalic nucleus by EOD or artificial stimuli, was recorded using two wires (80 µm diameter, insulated except at their tips) lowered through a small hole in the skull down to 1000–1200 micrometers from the brain surface, and attached with dental cement. The animals were first chronically implanted and then restrained in a narrow pen made of plastic mesh for few hours until the effects of anesthesia disappeared. Signals obtained from the brain (including the EOD artifact and the field potential response of the fast electrosensory pathway at the magnocellularis mesencephalic nucleus) were differentially amplified (gain ×1000), band-pass filtered (10–10000 Hz), and digitized with a Digidata 1322A. Interfering stimuli at the fish mean rate were applied and recorded in a similar way as in behavioral experiments

### Statistical procedures

We compared responsiveness to equal intensity and duration stimuli timed as the EOD or as the interference signals in a train copying the time sequence of a behavioral experiment using either the Wilcoxon signed-rank test (paired data) or the Wilcoxon rank-sum test. Two indicators were compared: failure rates, estimated by the percentage of stimuli that lack responses, and latency variability, estimated by the coefficient of variation of the latency). As a control experiment we made the same comparison when the same cell was stimulated by a train in which the inter EOD intervals were the same but its sequence was altered by shuffling. We compared histograms using the chi squared test.
